# Quantification of gastroesophageal regurgitation in brachycephalic dogs

**DOI:** 10.1111/jvim.16400

**Published:** 2022-04-07

**Authors:** Carla Appelgrein, Giselle Hosgood, Mary Thompson, Flaminia Coiacetto

**Affiliations:** ^1^ School of Veterinary Medicine Murdoch University Perth Western Australia Australia

**Keywords:** brachycephalic, digitrapper, reflux, regurgitation

## Abstract

**Background:**

Gastroesophageal reflux and regurgitation occurs in brachycephalic dogs, but objective assessment is lacking.

**Objectives:**

Quantify reflux in brachycephalic dogs using an esophageal pH probe and determine the association with scored clinical observations.

**Animals:**

Fifty‐one brachycephalic dogs.

**Methods:**

Case review study. Signs of respiratory and gastrointestinal disease severity were graded based on owner assessment. An esophageal pH probe with 2 pH sensors was placed for 18‐24 hours in brachycephalic dogs that presented for upper airway assessment. Proximal and distal reflux were indicated by detection of fluid with a pH ≤4. The median reflux per hour, percentage time pH ≤4, number of refluxes ≥5 minutes and longest reflux event for distal and proximal sensors were recorded. Association of preoperative respiratory and gastrointestinal grade, laryngeal collapse grade, and previous airway surgery with the distal percentage time pH ≤4 was examined using 1‐way ANOVA.

**Results:**

A total of 43 of 51 dogs (84%; 95% confidence interval 72‐92) displayed abnormal reflux with a median (range) distal percentage time pH ≤4 of 6.4 (2.5‐36.1). There was no significant association between the distal percentage time pH ≤4 and respiratory grade, gastrointestinal grade, laryngeal collapse grade, or previous upper airway surgery.

**Conclusions and Clinical Importance:**

The occurrence of reflux is not associated with owner‐assessed preoperative respiratory and gastrointestinal grade, laryngeal collapse grade, and previous airway surgery. Esophageal pH measurement provides an objective assessment tool before and after surgery.

## 1 INTRODUCTION

Gastroesophageal reflux disease is a multifactorial process characterized by failure of the normal anti‐reflux barrier protection against frequent and abnormal amounts of aboral flow of gastric contents or fluid.^1^ Clinical signs of reflux in dogs are thought to be gagging or retching, lip licking, extension of the neck after eating, eructating, or hiccups. However, identifying these events in dogs is challenging because of the absence of the dog—clinician communication that is crucial for the recognition of symptomatic reflux events in people. Instead, there is dependence on the attentiveness of owners, which is naturally confounded by inherent subjective interpretation of non‐specific clinical signs.^2^

Dogs with brachycephalic obstructive airway syndrome can have gastroesophageal reflux and regurgitation, but the pathogenesis is unknown. There is a relationship between the severity of digestive and respiratory clinical signs in brachycephalic dogs.^3^ It is theorized that the higher prevalence of regurgitation in brachycephalic dogs is, in part, due to the high negative intrathoracic pressures generated to overcome upper respiratory tract obstruction.^4^ The persistence of regurgitation after upper airway surgery in brachycephalic breeds is being noticed with increased frequency,^5^ with these dogs at risk of developing aspiration pneumonia. The potential presence of a sliding hiatal hernia is commonly held responsible.^6^, ^7^ However, recently the conformation of the esophageal hiatus in brachycephalic dogs has been evaluated and esophageal hiatal rim malformation likely plays a significant role in reflux and regurgitation.^8^, ^9^

Clinical evaluation of brachycephalic dogs presented for airway surgery routinely includes thoracic radiography, and a dynamic upper airway examination.^10^, ^11^ The need to investigate signs of gastrointestinal disease has been guided by the clinical signs observed by owners. Diagnostics have focused on identification of a hiatal hernia,^12^, ^13^ but no objective assessment of gastroesophageal reflux has been reported for brachycephalic dogs.

Twenty‐four‐hour ambulatory esophageal pH monitoring directly measures the extent and frequency of acid reflux into the esophagus and is the most sensitive and specific test to objectively diagnose gastroesophageal reflux in people.^14^ Catheter‐based pH monitoring uses 1 or more esophageal pH sensors mounted on a flexible catheter that is connected to a data storage device (Digitrapper pH‐Z Testing System, Medtronic, North Ryde, Australia), which continuously records pH in the esophagus. Monitoring pH allows for direct diagnosis of episodes of gastroesophageal reflux and quantifies the exposure of the esophagus to acid.^15^

The characterization of gastroesophageal reflux in brachycephalic dogs warrants further investigation. The purpose of this study was to objectively measure reflux in brachycephalic dogs using a catheter‐based pH sensor and determine the association with scored clinical observations of preoperative owner‐assessed respiratory and gastrointestinal grade, laryngeal collapse grade, and previous airway surgery. We hypothesized that reflux occurrence would be associated with preoperative respiratory and gastrointestinal grade, laryngeal collapse grade, and previous airway surgery.

## 2 MATERIALS AND METHODS

Ethical consent was obtained from Murdoch University (R3116/19) for the study and all owners consented to enrolment of their dog. The frequency of signs of upper respiratory disease (snoring, inspiratory effort, exercise, or stress intolerance, and syncope) and signs of gastrointestinal disease (eg, vomiting, regurgitation, gagging or retching, lip licking, extension of neck after eating, burping, or hiccups) was recorded using an owner questionnaire (Appendix S1). Scoring of signs of respiratory and gastrointestinal disease was based on the scheme used by Poncet et al.^3^, ^16^ Each sign of respiratory and gastrointestinal disease was classified according to frequency: absent or occasional (category 1), daily (category 2), or more than once daily (category 3) and the overall respiratory or gastrointestinal grade defaulted to the highest classification of any 1 clinical sign.

The dogs were admitted for placement of a catheter‐based esophageal pH monitor (Digitrapper pH‐Z Testing System, Medtronic, North Ryde, Australia). This is an ambulatory pH‐recording device, which consists of a 70‐cm long, 2.1‐mm diameter, flexible probe connected to an external reader. The probe contained 2 recording sensors, 15 cm apart, designed to detect proximal and distal reflux. The electrodes were calibrated to a reference solution of pH 1 and 7 before placement, as per the manufacturer's instructions.

**FIGURE 1:**
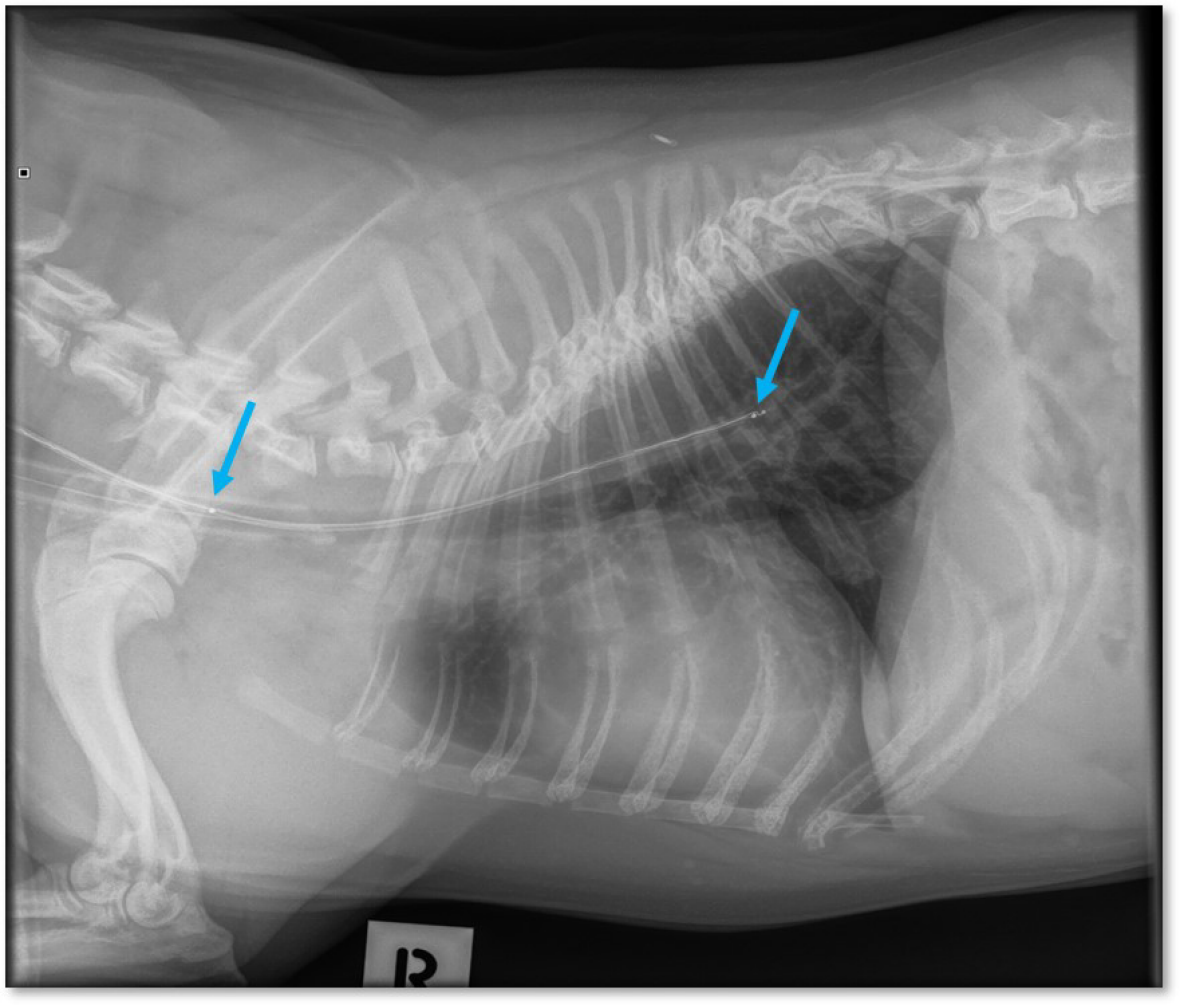
Right lateral thoracic radiograph of a 1‐year‐old French bulldog showing the position of the esophageal probe with the proximal and distal sensor location (arrows)

**FIGURE 2:**
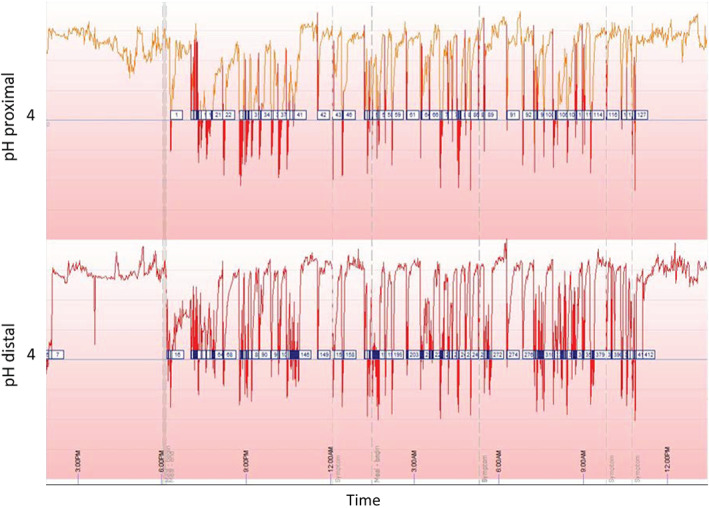
Typical pH trace of a 7‐year‐old French bulldog with 5.4 regurgitation/h recorded at the proximal sensor and 17.4 reflux/h recorded at the distal sensor during a 24‐hour period. The percentage time pH < 4 was 10.6% and 12.6% at the proximal and distal sensors, respectively

Food was withheld for 12 hours before admission. Dogs were sedated with butorphanol (0.3 mg/kg IM; Troy laboratories, Glendenning, NSW, Australia) and medetomidine (5 μg/kg IM; Troy laboratories, Glendenning, NSW, Australia). All dogs underwent total intravenous anesthesia using Alfaxan to effect (1‐2 mg/kg IV; Jurox, Rutherford, NSW, Australia). Dogs were intubated with an endotracheal tube to secure and protect the airway and were provided with oxygen supplementation. Intra‐nasal lidocaine20 was administered and (1‐2 mg/kg intranasal; Troy laboratories, Glendenning, NSW, Australia) the esophageal pH probe was inserted through 1 nostril into the esophagus. The distal sensor was positioned at the level of the 8th rib (Figure 1), confirmed by 3‐view thoracic radiographs. Once in place, the esophageal pH probe was secured to the alar fold with non‐absorbable suture (3/0 Nylon) in a Chinese finger trap pattern. Atipamezole (0.25 mg/kg; Troy laboratories, Glendenning, NSW, Australia) was administered and the dogs recovered under close observation. When the device was in place, recording began and continued for 24 hours. An esophageal reflux event was defined as a single pH measurement ≤4.^17^ Any reflux event that occurred while the dog was still sedated was excluded from analysis. The computer software (AccuView pH‐Z 5.2 software) generated a pH trace (Figure 2), and the variables were calculated by the computer software (AccuView pH‐Z 5.2 software) for the proximal and distal probe, including the duration of the study, reflux per hour, number of reflux ≥5 minutes, duration of longest reflux (minutes), and the percentage time pH ≤4 for both sensors. The data reader was attached to the dog's collar or the cage. All dogs remained hospitalized for the study period and followed a typical daily routine (eg, feeding regimen, walking) with the time of food intake recorded. Food offered in hospital was consistent with that fed at home.

After the 24‐hour esophageal pH recording, all dogs underwent upper airway examination. The pharynx and larynx were examined and the stage of laryngeal collapse was recorded.^18^ No premedication was administered but Alfaxan (3 mg/kg, IV to effect; Troy laboratories, Glendenning, NSW, Australia) was administered to induce anesthesia. The evaluator's assessment of arytenoid abduction activity was facilitated by the anesthetist verbally indicating the onset of each inspiration.

Upper gastrointestinal endoscopy was performed in the first 10 dogs enrolled.^19^ The placement of the esophageal pH probe was visualized and its relation to the gastroesophageal junction was noted. Mucosal biopsies (5 mm) were collected from the distal esophagus, cardia, body of the stomach, pyloric antrum, and duodenum, in some dogs. The specimens were fixed in 10% buffered formalin and processed for later histologic examination according to WSAVA guidelines by a board‐certified pathologist (FC).^19^

Dogs underwent surgical intervention as indicated by their clinical presentation and diagnostic results. Surgical intervention performed included sutured staphylectomy, wedge or Trader's alarplasty, tonsillectomy, and circumferential hiatal rim reconstruction as described.^9^

Owners were asked to return their dogs 6 months after surgical intervention for esophageal monitoring. The esophageal pH probe was replaced as described and monitored for 24 hours.

### 2.1 Reflux variable standardization

The percentage time pH ≤4 at the distal probe provides an overall summation of the variables measured by the esophageal pH probe, reflecting both the number of events, and duration of events. For each dog (*i*), the standardized score of the distal percentage time pH ≤4 was calculated against the mean and SD for the respective variable from 21 normal dogs (*x*) as reported by McMahon.^20^

$$\text{Standardized parameter score}\;\left(i\right)=\frac{\left({\text{Measurement}}_{i}-{\text{mean}}_{x}\right)}{{\mathrm{SD}}_{x}}$$

Based on the Gaussian distribution of the variable for normal dogs in McMahon's study, 95% of measurements for all normal dogs would be contained within ± 1.96 SDs of the mean_*x*_, thus, a variable score >2 would be outside the upper reference limit and considered abnormal.^21^ This is akin to documenting whether or not a dog is above the outer limit of the reference interval, which is established from the mean ± 1.96 SD.^22^ Any dog below this limit would be within the reference range, or below the lower limit, which is irrelevant in this context. The distal percentage time pH ≤4 score was used to discriminate normal from abnormal dogs for the purpose of reporting the summary data for the pH monitoring; dogs with a score of ≤2 were considered normal and >2 were considered abnormal.

### 2.2 Statistical analysis

Data were tested for normality before summarization with most data highly skewed and thus numerical data describing the cohort were summarized and reported as median and range. The frequency of categorical data was reported, and any proportions are presented as a percentage with a 95% confidence interval (CI) of the percentage. The response of interest for statistical analysis was the standardized score for the distal percentage time pH ≤4, which was transformed (square root) to follow a normal distribution. The association of each categorical (ordinal) explanatory variable (preoperative respiratory and gastrointestinal grade, laryngeal collapse grade, and previous airway surgery) with the standardized score was examined using 1‐way ANOVA with significance considered at *P* ≤ .05. We hypothesized that the standardized score would be associated with the preoperative respiratory and gastrointestinal grade, laryngeal collapse grade, and previous airway surgery. The proportion of variance in the distal percentage time pH ≤4 explained by the examined variables was recorded by the coefficient of determination (*R* ^2^). SAS v 9.4 (SAS Institute, Cary, NC) was used for all analyses.

## 3 RESULTS

From June 2019 to May 2021, 51 brachycephalic dogs (25 females, 26 males) qualified for inclusion. There were 35 French bulldogs, 7 bulldogs, 6 pugs, and 3 Boston terriers. The median age was 1.6 years (range, 0.4‐8). Based on owner assessment, signs of respiratory disease were grade 1 in 12 dogs (24%; 95% CI 14‐37), grade 2 in 9 dogs (18%; 95% CI 10‐30), and grade 3 in 30 dogs (59%; 95% CI 45‐71). Signs of gastrointestinal disease were grade 1 in 22 dogs (43%; 95% CI 31‐57), grade 2 in 15 dogs (29%; 95% CI 19‐43), and grade 3 in 14 dogs (27%; 95% CI 17‐41).

Eight dogs (16%; 95% CI 8‐28) were considered normal with a median (range) distal % time pH ≤4 of 0.3 (0‐2.4) and 43 dogs (84%; 95% CI 72‐92) considered abnormal with a median (range) distal % time pH ≤4 of 6.4 (2.5‐36.1) based on a distal percentage time pH ≤4 with a score ≤2 or >2, respectively. The summary statistics of the measured variables reflect this division with higher values for abnormal dogs across all variables measured (Table 1).

**TABLE 1:** Summarized measurements of reflux from esophageal pH probes with proximal and distal sensors placed in 51 brachycephalic dogs

	Reference (n = 21)^21^	Normal (n = 8)	Abnormal^a^ (n = 43)
	Mean (SD)	Median (range)	Median (range)
Study duration (h)	24	23.2 (18–24)	22 (17‐25)
Proximal percentage time pH ≤ 4		0 (0‐0.4)	2.8 (0.1‐14)
Distal percentage time pH ≤ 4	0.43 (0.86)	0.3 (0‐2.4)	6.4 (2.5‐36.1)
Proximal reflux/h		0.1 (0‐0.8)	1.9 (0.2‐6.8)
Distal reflux/h	0.3 (0.55)	0.4 (0‐2.1)	3.8 (0.9‐49.4)
Proximal number of refluxes >5 min		0	1 (0‐6)
Distal number of refluxes >5 min	0.24 (0.54)	0 (0‐2)	2 (0‐22)
Proximal duration of longest reflux (min)		0 (0‐2)	9 (0‐60)
Distal duration of longest reflux (min)	2.52 (4.04)	2 (0‐21)	20 (2‐120)

^a^ Abnormal defined as % time pH ≤4 score >2.

Based on 3 view thoracic radiographs, there was no evidence of pulmonary lesions, but a hiatal hernia was detected in 1 dog. Seven dogs (14%; 95% CI 7‐26) had previous airway surgery. On upper airway examination, laryngeal collapse was not identified in 2 dogs (4%; 95% CI 2‐13) and was identified as grade 1 in 12 dogs (24%; 95% CI 14‐37), grade 2 in 22 dogs (43%; 95% CI 31‐57), and grade 3 in 15 dogs (29%, 95% CI 19‐43).

Endoscopy was performed in the first 10 dogs enrolled. Esophagoscopy showed mild (4/10 dogs, 40%; 95% CI 17‐69), moderate (4/10 dogs, 40%; 95% CI 17‐69), and severe (2/10 dogs, 20% 95% CI 6‐51) mucosal lesions. During examination, 8 of 10 dogs had an open gastroesophageal junction, and 2 dogs had a closed gastroesophageal junction. Endoscopy confirmed the hiatal hernia identified on thoracic radiographs in 1 dog. The distal pH sensor was visualized 5 cm proximal to the gastroesophageal junction in all dogs. Two dogs underwent esophageal biopsy and histologic examination revealed normal findings in 1 dog and mild keratinization in the other. Gastroscopy showed no (7/10 dogs, 70%; 95% CI 40‐90) and mild (3/10 dogs, 30%; 95% CI 11‐60) mucosal lesions. Gastric biopsy revealed mild fibrosis/glandular nesting/mucosal atrophy in 4 of 10 (40%; 95% CI 17‐69) dogs, mild lamina propria lymphocyte and plasma cell infiltration in 6 of 10 dogs (60%; 95% CI 31‐83), and mild lamina propria neutrophil infiltration in 5 of 10 dogs (50%; 95% CI 24‐76). Five dogs showed multifocal, minimal, patchy inflammatory changes and 4 dogs had no histologic changes.

Forty‐four dogs underwent a sutured staphylectomy, wedge or Trader's alarplasty, and tonsillectomy; the remaining 7 dogs had undergone previous airway surgery. Thirty‐six dogs underwent an exploratory celiotomy and circumferential, esophageal hiatal rim reconstruction. All these dogs had a lax phrenico‐esophageal ligament and enlarged esophageal hiatus noted at surgery, with the surgeon able to pass 3 or more fingers through the esophageal hiatus.^9^

Seven dogs that had undergone esophageal hiatal rim reconstruction, staphylectomy, and alarplasty returned 6 months postoperatively for esophageal pH probe monitoring. All 7 dogs (100%; 95% CI 60‐100) had a distal percentage time pH ≤4 score above 2 preoperatively with a median (range) distal % time pH ≤4 of 6.2 (5.1‐36.1). Signs of respiratory disease were grade 2 in 3 dogs and grade 3 in 4 dogs. Signs of gastrointestinal disease were grade 1 in 1 dog, grade 2 in 4 dogs, and grade 3 in 2 dogs. Following esophageal hiatal rim reconstruction, staphylectomy, and alarplasty, all 7 dogs (100%; 95% CI 60‐100) showed marked improvement postoperatively with minimal to no reflux detected (Table 1) and a distal percentage time pH ≤4 score below 2 (Table 2).

**TABLE 2:** Preoperative and postoperative (6 months) summarized measurements of reflux from esophageal pH probes with proximal and distal sensors placed in 7 brachycephalic dogs undergoing circumferential esophageal hiatal rim reconstruction, staphylectomy, and alarplasty

	Reference (n = 21)^21^	Preoperative (n = 7)	Postoperative (n = 7)
	Mean (SD)	Median (range)	Median (range)
Study duration (h)	24	23.25 (22‐23.5)	24.2 (24‐26)
Proximal percentage time pH ≤4		4.7 (0.9‐13.3)	0
Distal percentage time pH ≤4	0.43 (0.86)	13 (5.1–36.1)	0.1 (0‐0.5)
Proximal reflux/h		3.8 (1.4‐6.8)	0 (0‐0.2)
Distal reflux/h	0.3 (0.55)	5.6 (1.4‐49.4)	0.05 (0‐0.5)
Proximal number of refluxes >5 min		2.5 (0‐6)	0
Distal number of refluxes >5 min	0.24 (0.54)	7.5 (1‐22)	0 (0‐1)
Proximal duration of longest reflux (min)		12.5 (2‐34)	0
Distal duration of longest reflux (min)	2.52 (4.04)	28 (12‐72)	0

There was no significant association between the standardized score for distal % time pH ≤4 and respiratory grade (*P* = .17; *R* ^2^ = −.08), gastrointestinal grade (*P* = .63; *R* ^2^ = .02), laryngeal collapse grades (*P* = .62, *R* ^2^ = .02), or whether the dog had previous upper airway surgery (*P* = .46, *R* ^2^ = .01).

## 4 DISCUSSION

Results of this study identified a substantial number and duration of reflux events in this cohort of hospitalized brachycephalic dogs. While distal reflux events will predispose dogs to esophagitis, events at the proximal sensor will predispose dogs to regurgitation, aspiration, and pneumonia. Furthermore, persistent regurgitation might aggravate signs of respiratory disease by inducing inflammation in the pharyngeal region.^23^ Symptomatic reflux events in people with abnormal acid exposure are of longer duration and to a higher proximal extent compared to asymptomatic reflux episodes.^24^ Based on the findings of our study, we fail to reject the null hypothesis that the occurrence of reflux is not associated with preoperative respiratory and gastrointestinal grade, laryngeal collapse grade, and previous airway surgery.

In people, the percentage time pH ≤4 has been established as an accurate indicator of gastroesophageal reflux disease.^25^ Assessment can also be based on the DeMeester score, a composite score derived from 6 variables: percent of time the esophageal pH ≤4 in total, and in the upright and supine positions, total number of reflux episodes, number of reflux episodes lasting ≥5 minutes, and the duration of the longest episode.^17^, ^26^ The individual variable scores are calculated as a Z‐score.^27^ However, composite scores have the disadvantage of weighting components equally, and potentially diluting out effects of individual variables or over emphasizing a result. Since the variables are not independent, the composite score can be misleading. Given that the distal percentage time pH ≤4 reflects the number and duration of events, we focused on this score for evaluation. Assessment of the number of reflux episodes lasting ≥5 minutes and the duration of the longest episode are important as these reflect the duration the esophagus is exposed to an acidic environment,^17^, ^26^ but again, are encompassed in the percentage time pH ≤4. For interpretation, we chose to standardize the variable for ease of assessment and purposely avoided a composite score. The standardized score of a dog is the Z‐score, indicating how many SDs the dog's value is away from the expected mean of the normal population; in this case, only exceeding 2 SDs is relevant.^27^ Thus, a score greater than 2 indicates the dog's value is outside of the upper extreme of 95% of the normal population (2 SDs) and could therefore be interpreted as abnormal.^21^ Only reference values from previous experimental work in canine models are currently available for the distal sensor location. While events to the proximal sensor may pose aspiration risk, events at the distal sensor are more frequent and represent exposure of the gastroesophageal sphincter to an acid environment, which can cause mucosal damage and self‐perpetuate the reflux.^28^ We are collating reference values from a cohort of healthy dogs without gastrointestinal disease, under environmental and assessment conditions that mimic those of this current cohort.

The distal percentage time pH ≤4 provides an overall summation of the variables measured by the esophageal pH probe and was the focus of our statistical analysis.^25^ This variable encompasses number of events and durations of events. We identified a median of 6.4% duration at the distal sensor for all dogs, which is substantially higher than the reported reference of 0.43%.^20^ The presence of gastroesophageal reflux and subsequent esophagitis have been identified as being responsible for shortening of lower esophageal sphincter and further decreasing its pressure.^28^ thereby causing self‐perpetuating reflux. In addition to noting the occurrence of reflux in a healthy cohort of 21 dogs, McMahon et al surgically induced a hiatal hernia in 18 dogs. In these 18 dogs, the distal percentage time pH ≤4 ranged from 3.6‐29% with a mean (SD) of 13.8% (7.4).^20^ In people, a distal percentage time pH ≤4 above 5% is considered pathologic.^29^ Thus, our results are consistent with abnormal reflux.

The distal percentage time pH ≤4 score was not explained by the preoperative gastrointestinal grade, as identified by owners. This indicates that owners are unable to identify the severity of the problem, possibly because it is a silent disease, or because any clinical signs that are seen are not as reflective of the events as we might think. Additionally, stress and or excitement under hospital conditions may have exacerbated reflux episodes. Endoscopy performed in 10 dogs did identify changes consistent with esophagitis and this indicates that the reflux identified by the pH monitoring did have pathophysiologic consequences. In people, a symptom index is calculated for use in clinical decision‐making. People press a button on the recording device when symptoms such as heartburn and indigestion are perceived. The number of reflux‐related symptomatic episodes is divided by the total number of symptomatic episodes and expressed as a percentage.^30^ Dogs were hospitalized for the duration of placement of the esophageal probe but despite close observation, it was not possible to consistently note when suspected reflux episodes were seen. This is further compromised by the absence of dog‐to‐clinician communication since dogs cannot relay symptoms (a perceived feeling). Future research is investigating 24‐hour video monitoring to detect any consistent observed behavior coinciding with reflux events identified by the esophageal pH probe.

The distal percentage time pH ≤4 score was also not explained by the preoperative respiratory grade, laryngeal collapse grade, or whether dogs had undergone previous airway surgery. Laryngeal collapse develops secondary to the turbulent airflow and chronic high negative pressures in the pharynx of brachycephalic dogs.^11^ We included this variable to reflect the presence of high negative pressures generated to overcome upper respiratory tract obstruction. If the theorized higher prevalence of regurgitation in brachycephalic dogs was, in part, due to the high negative intrathoracic pressures generated to overcome upper respiratory tract obstruction,^4^ then an association between the degree of reflux and laryngeal collapse would be expected. Historically, the prevalence of hiatal hernia in dogs with brachycephalic obstructive airway disease has been cited as low compared to the high prevalence of gastrointestinal clinical signs identified in the population.^3^, ^13^, ^16^ In our cohort, a hiatal hernia was only diagnosed preoperatively in 1 dog despite the high number of reflux events recorded in most dogs. Preoperative detection of a hiatal hernia may have been increased by performing additional manipulations during thoracic radiographs or endoscopy, as described by Broux et al^13^ but we deem it unlikely that we would have diagnosed a hiatal hernia in all dogs. This supports the notion that reflux may be the result of other features such as the conformation of the esophageal hiatus,^8^, ^9^ and not only be a consequence of a hiatal hernia. We suspect that hiatal hernia is a late consequence of abnormal hiatal rim conformation and laxity, and that substantial reflux may occur well before a hiatal hernia is ever present or documented.

Reflux episodes are characterized by an abrupt decline in intra‐esophageal pH in the esophagus. A cut‐off at pH ≤4.0 was used to identify acid reflux episodes since the proteolytic activity of pepsin rapidly decreases in solutions with a pH > 4.0^31^ and typical reflux symptoms (ie, heartburn) are more often reported in people at intra‐esophageal pH values ≤4. In addition, recent animal data have strongly implicated the proteolytic enzyme pepsin as significant injurious in hemorrhagic erosive esophagitis^32^ such that inactivation of this enzyme by raising intraluminal pH > 5 becomes clinically relevant.^31^

To facilitate placement of the esophageal pH probe, dogs underwent total intravenous anesthesia. This was required as the pH probe is soft and can be difficult to place in the conscious dog without the ability to visualize it in the pharynx. We elected to intubate dogs to secure the airway to limit the risk of aspiration pneumonia and to facilitate correct placement of the probe into the esophagus. The agents used have not been specifically investigated in dogs and we can find no evidence on whether they alter the propensity for gastroesophageal reflux. In people, deep sedation has no effect on reflux episodes^33^ and Garcia^20^ found no association between the use of opioid drugs and gastroesophageal reflux in people. Butorphanol was used for its sedative effects and short duration of action.^34^ Medetomidine was used since it is reversible.^34^ Alfaxalone was used since it is short‐acting.^35^ Inhalation anesthetic agents were not used as they are associated with a risk for gastroesophageal reflux in dogs.^36^ The first reflux event identified was excluded from analysis since it may have occurred during anesthetic recovery. Research is currently evaluating this anesthetic protocol in non‐brachycephalic dogs without a history of gastrointestinal disease to determine the effect on gastroesophageal reflux.

A total of 37 dogs underwent exploratory celiotomy to examine the conformation of the esophageal hiatus based on their history and results from the esophageal pH probe monitoring. Given the role of the esophageal hiatus in creating a pinch‐cock effect at the level of the gastroesophageal junction, we propose that dogs with substantial reflux will benefit from esophageal hiatal rim reconstruction to enhance this effect.^9^, ^37^ Vindication of the decision to explore and reconstruct the esophageal hiatal rim may be best determined by reflecting on the objective assessment of the esophageal pH monitoring in light of the clinical picture. In the dogs that returned for assessment 6 months after esophageal hiatal rim reconstruction, reflux was markedly reduced based on objective re‐assessment using the pH probe. Of note too, is that these dogs were not on medication, indicating that the surgical intervention was useful in these cases where reflux was persistent.

In conclusion, the occurrence of reflux identified using esophageal pH measurement was not explained by the preoperative respiratory grade or gastrointestinal grade, laryngeal collapse grade, or by previous upper airway surgery. Thus, owner observation did not recognize the severity of the problem. Furthermore, the degree of respiratory compromise, reflected by laryngeal collapse, does not play a dominating role and previous airway surgery did not appear to have a mitigating effect. Catheter‐based esophageal pH measurement is a minimally invasive diagnostic tool to objectively assess the occurrence of reflux. Esophageal pH measurement provides an objective assessment tool before and after surgery.

## CONFLICT OF INTEREST DECLARATION

Authors declare no conflict of interest.

## OFF‐LABEL ANTIMICROBIAL DECLARATION

Authors declare no off‐label use of antimicrobials.

## INSTITUTIONAL ANIMAL CARE AND USE COMMITTEE (IACUC) OR OTHER APPROVAL DECLARATION

Approved by Murdoch University (R3116/19).

## HUMAN ETHICS APPROVAL DECLARATION

Authors declare human ethics approval was not needed for this study.

## Supporting information


**Appendix S1**. Supporting Information
