# Supplementary material for: Quantification of gastroesophageal regurgitation in brachycephalic dogs
Source: J Vet Intern Med. 2022 Apr 7;36(3):927–34. doi: 10.1111/jvim.16400 (PMC9151495; doi:10.1111/jvim.16400)
Supplement: Supplementary file 1 — Appendix S1. Supporting Information [file JVIM-36-927-s001.pdf]

Small Animal Referral

Dog Name: ____________________ **Breed:** ________________________________

Age: _______________ **Period of ownership:** ___________________

Respiratory	Frequency (sporadic – frequent)				
**Does your dog do this?**	Never	Weekly	Twice a week	Daily	Multiple times daily
Noisy breathing					
Snore					
Pant excessively					
Flop on the floor after exercise					
Have difficulty sleeping					

Gastrointestinal	Frequency (sporadic – frequent)					Assoc. with Exercise / excitement	
**Does your dog do this?**	Never	Weekly	Twice a week	Daily	Multiple times daily	**Yes**	**No**
Regurgitate							
Vomit							
Gag							
Retch							
Lip lick							
Extend neck after eating							
Burp							
Hiccup							
Periodic diarrhoea							

At what age did the gastrointestinal signs start? __________

What food are you currently feeding your dog? ____
